# Advances in gold catalyzed synthesis of quinoid heteroaryls

**DOI:** 10.1039/d4ra03368j

**Published:** 2024-07-03

**Authors:** Adnan Majeed, Ayesha Zafar, Zanira Mushtaq, Muhammad Adnan Iqbal

**Affiliations:** a Department of Chemistry, University of Agriculture Faisalabad Faisalabad-38000 Pakistan adnan.iqbal@uaf.edu.pk; b Organometallic and Coordination Chemistry Laboratory, University of Agriculture Faisalabad Faisalabad-38000 Pakistan

## Abstract

This review explores recent advancements in synthesizing quinoid heteroaryls, namely quinazoline and quinoline, vital in chemistry due to their prevalence in natural products and pharmaceuticals. It emphasizes the rapid, highly efficient, and economically viable synthesis achieved through gold-catalyzed cascade protocols. By investigating methodologies and reaction pathways, the review underscores exceptional yields attainable in the synthesis of quinoid heteroaryls. It offers valuable insights into accessing these complex structures through efficient synthetic routes. Various strategies, including cyclization, heteroarylation, cycloisomerization, cyclo-condensation, intermolecular and intramolecular cascade reactions, are covered, highlighting the versatility of gold-catalyzed approaches. The comprehensive compilation of different synthetic approaches and elucidation of reaction mechanisms contribute to a deeper understanding of the field. This review paves the way for future advancements in synthesizing quinoid heteroaryls and their applications in drug discovery and materials science.

## Abstract 2

Synthesis of quinoid heteroaryls *via* gold-catalyzed cascade protocols: recent advances and methodologies.

## Graphical abstract

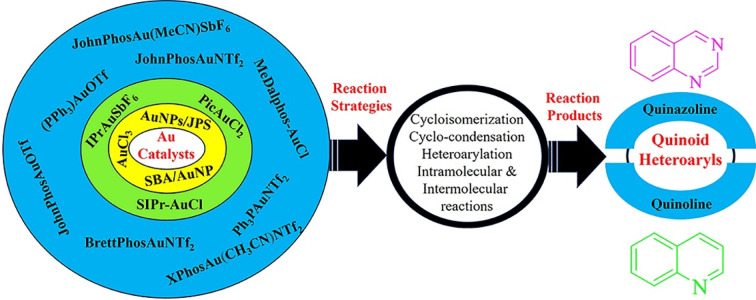

## 1. Introduction

Over the past two centuries, significant research attention has been directed towards quinoid alkaloids, spurred by the isolation of quinine from cinchona tree bark in 1820 and the vaccine from *Adhatoda vasica* in 1888. These classes have yielded over 600 alkaloids, showcasing diverse biological activities.^1–7^ Many of these compounds have played crucial roles in medicinal chemistry, materials science, and optoelectronics.^8,9^ Traditional synthetic methods for quinoline and quinazoline derivatives often face challenges such as harsh conditions, limited substrates, multistep processes, and waste generation, necessitating innovative approaches.^10^ Transition metal^11^ catalyzed formation of N-heterocycles remains a vibrant research area due to the metal's electron transfer capabilities, availability, and efficiency as catalysts. These reactions offer mild conditions and compatibility with various functional groups, making them highly desirable for organic transformations.^12–14^

Because gold has special catalytic properties and is the most electronegative metal in Pauling's scale, it is chosen as a catalyst over other transition metals,^15^ underscoring its distinctiveness and importance in catalysis. Advances in homogeneous and heterogeneous catalysis techniques have made gold catalysis a “hot topic” in the realm of organic synthesis. The pioneering work of Hutchings and Haruta in the 1980s laid the foundation for heterogeneous gold catalysis, demonstrating its efficacy in acetylene hydrochlorination and CO oxidation. This catalytic system, characterized by gold nanoparticles supported on various substrates, has found extensive use in industrial processes due to its robustness and efficiency.^16^

Homogeneous gold catalysis had a rise in popularity in the 2000s because of its many reactivities, large selection of gold complexes, simplicity of usage, and moderate reaction conditions. Because of its adaptability, homogeneous gold catalysis has become an important synthetic tool for scientists studying materials, organic,^17^ and organometallic chemistry. This approach offers unparalleled control over reaction conditions and selectivity, making it an indispensable tool for synthetic chemists across diverse disciplines.^15,18–25^

Over recent years, gold carbene intermediates formed when an electrophile approached the distal end of an alkenyl gold complex, leading to various transformations,^26^ and making gold catalysis highly versatile. The gold-catalyzed generation of gold carbenes from readily available alkynes represents a major advancement in metal carbene chemistry, enhancing the scope and versatility of gold catalysis.^27^ Similarly, in organic synthesis, α-oxo metal carbenes/carbenoids^28^ played a crucial role in enabling complex processes such as cyclopropanation, ylide production, and C–H insertion.^29,30^ The protodeauration mechanism of various organogold compounds, including gold-alkyl,^31^ gold-alkynyl, and gold-allyl species, was also studied.^28,32,33^ A synergistic gold–iron and gold–palladium^34^ catalytic system enabled efficient C–C bond formation and macrocyclization under mild conditions, achieving up to 95% yields with excellent regioselectivity.^35^ Through gold-catalyzed alkyne hydroboration, a new class of stable four-coordinated benzotriazole-borane compounds was synthesized. These compounds exhibit intense fluorescence emission and great stability, making them suitable probes for use in the future.^36,37^

**Fig. 1:**
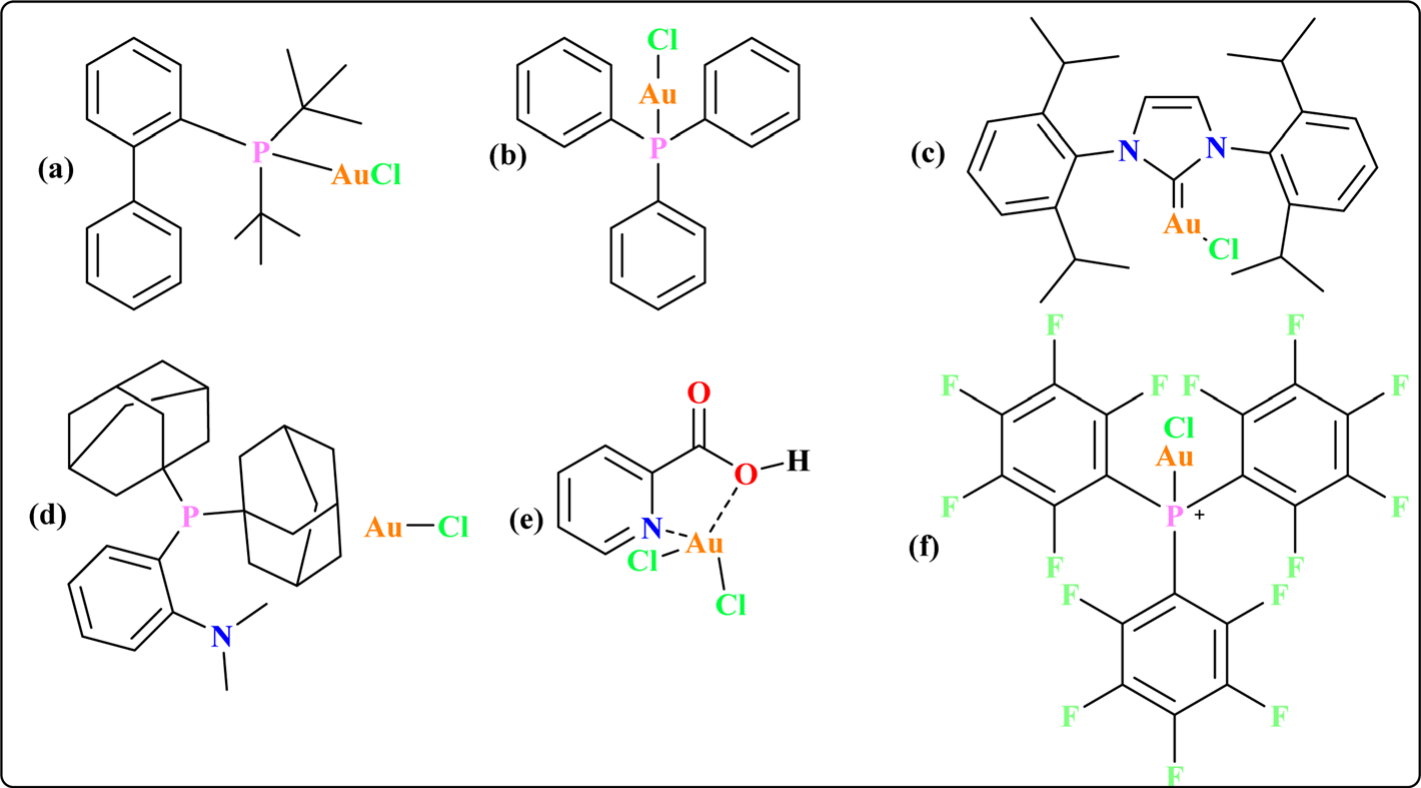
The structures of some gold catalysts (a–f) aid quinoid heteroaryl synthesis.

Some gold catalysts are shown in Fig. 1 which are used to synthesize quinoid heteroaryls. Acting as carbophilic π-Lewis acids, gold catalysts effectively trigger the activation of C–C multiple bonds, leading to the formation of reactive intermediates that facilitate subsequent reactions with diverse partners.^38^ In particular, enol/enamine-type reactive species are spontaneously formed *in situ* when these activated C–C multiple bonds contact with heteronucleophiles, allowing for the stimulation of several cascade cyclization events.^39–41^ Previous reviews by our group provided a comprehensive overview of the synthesis pathways for gold complexes^42^ and their versatile applications as anti-cancer agents across various therapeutic modalities.^43^ The recent review underscores the synthesis of quinoid heteroaryl using gold-catalyzed cascade protocols, emphasizing the need to enhance efficiency, expand substrate diversity, and investigate sustainable approaches. Collaboration between synthetic chemists and pharmaceutical researchers is essential for leveraging these advancements in drug discovery.

## 2. Gold catalyzed quinazoline reactions

### 2.1. Synthesis of pseudorutaecarpine (1b)

**Scheme 1:**
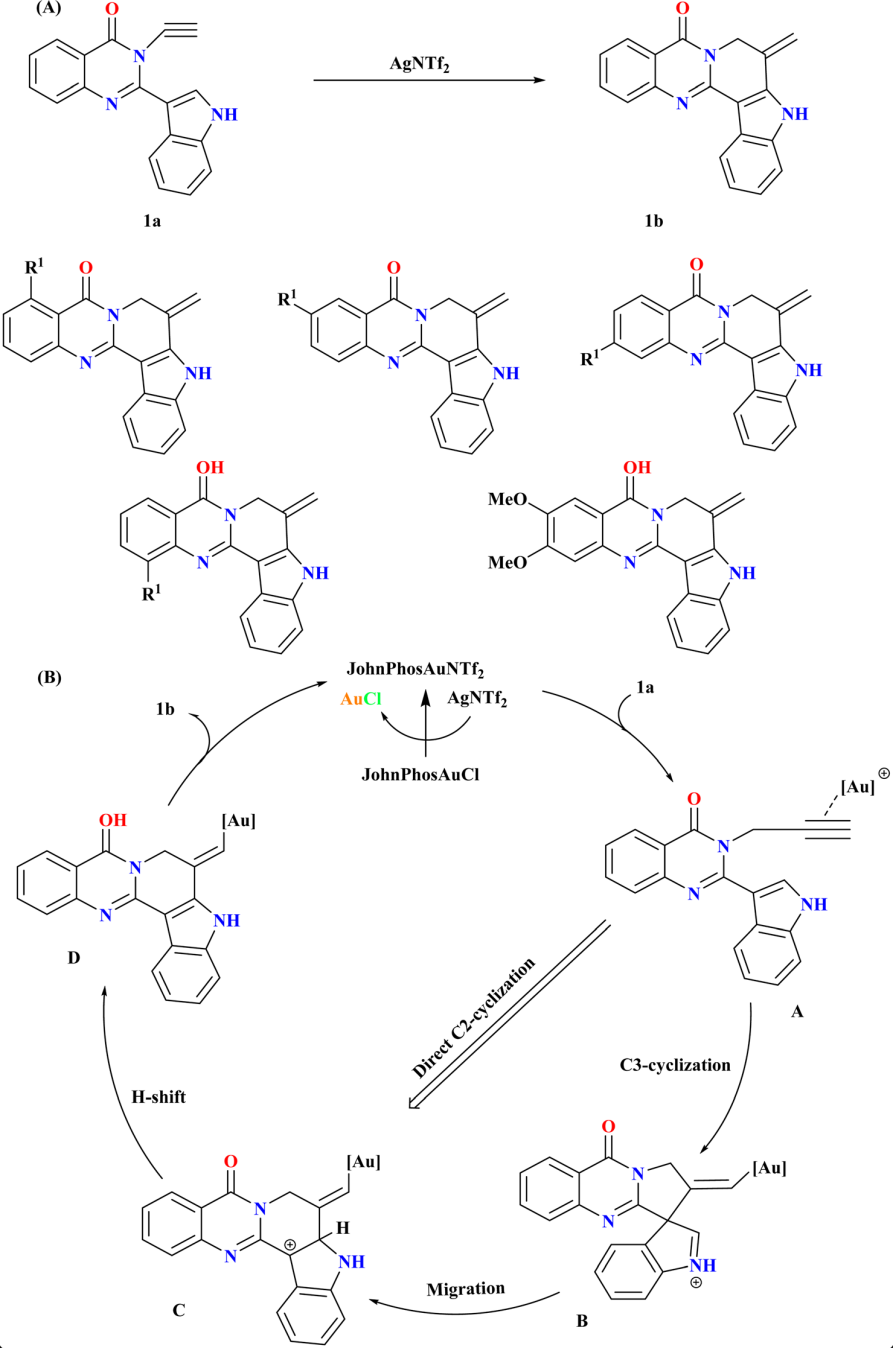
(A) Gold-catalyzed synthesis of 1b, (B) proposed reaction mechanism for synthesis of 1b.

Rutaecarpine derivatives, featuring quinazolinone and indole motifs, exhibit unique connectivity, notably in pseudorutaecarpine where quinazolinone C2 links to indole C3, a rare phenomenon in literature.^44–47^ Developing efficient synthetic methods and assessing their biological activities is crucial for exploring their potential pharmacological applications.^48^ Wang *et al.*, were synthesized numerous derivatives of pseudorutaecarpine with high yields using a gold-catalyzed selective cyclization and 1,2-shift of *N*-alkynyl quinazolinone-tethered indoles (Scheme 1A). As the model substrate, *N*-alkynyl quinazolinone-tethered indole (1a) was chosen, and at room temperature, it selectively produced pseudorutaecarpine (1b). Optimizing ancillary gold ligands revealed JohnPhos as highly effective (96% yield). Employing AgNTf_2_ as the Ag(i) salt and CH_3_CN as a solvent alongside JohnPhosAuCl significantly enhanced yield (92%). The sole use of gold did not yield 1b, affirming the necessity of both catalysts.^49^

Based on previous studies,^50–52^ the reaction mechanism for the formation of 1b from 1a involves a cationic gold-catalyzed complex formation activating the alkyne group to yield intermediate A^50,52,53^ as shown in (Scheme 1B). Intermediate B is formed by a subsequent 5-*exo*-dig cyclization that yields iminium/vinyl gold. Intermediate C was produced by a 1,2-shift that yields carbon cation. The catalytic cycle was finished when intermediate D produced pseudorutaecarpine 1b through proton removal and proton-deauration. An analogous route might be reached by directly C2-cyclizing intermediate A.^49^

### 2.2. Synthesis of functionalized quinazoline 1 oxide (1e)

**Scheme 2:**
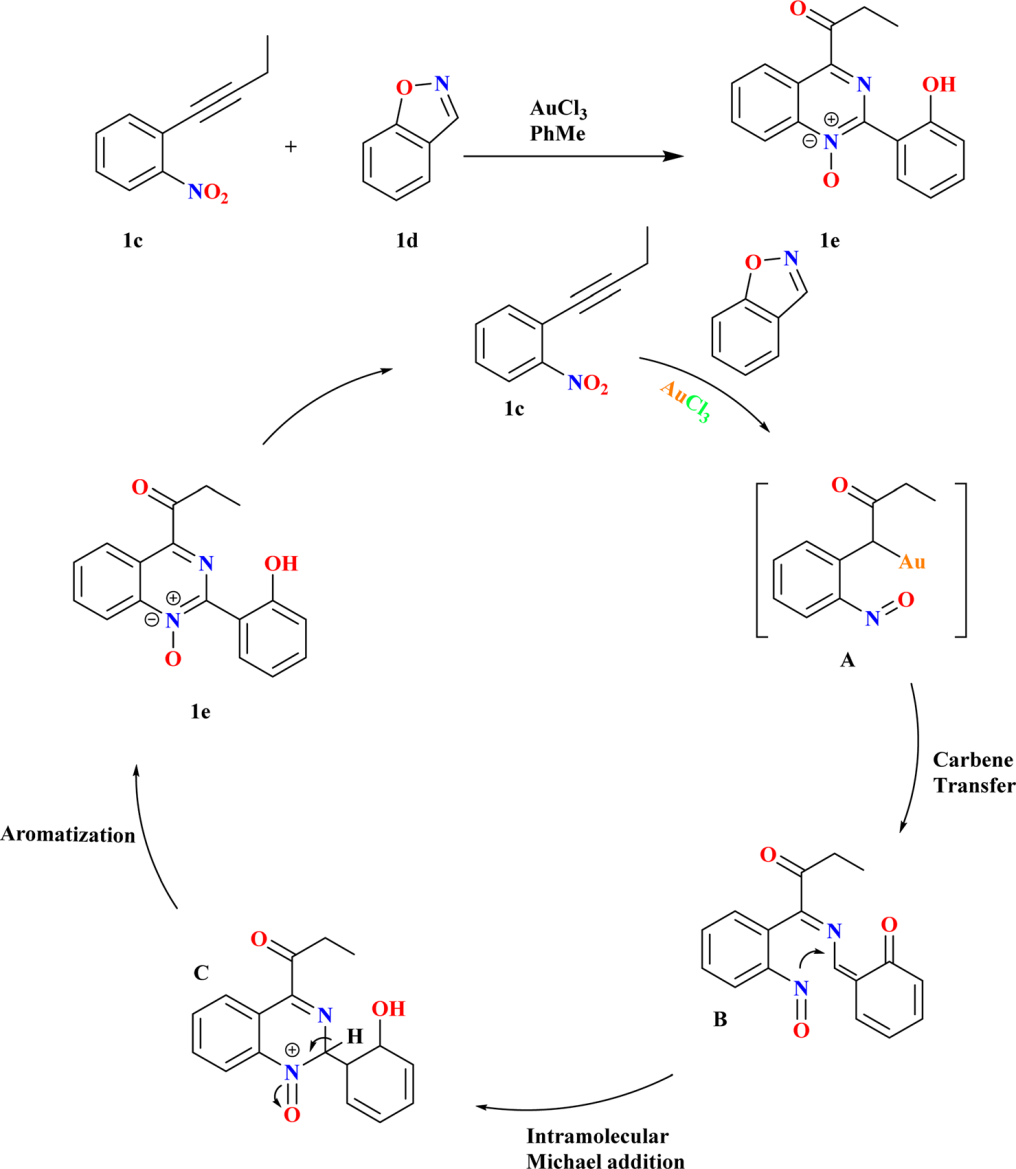
Gold-catalyzed synthesis of quinazoline 1e oxide.

Quinazoline 1-oxides remain underexplored in medicinal chemistry despite the parent quinazoline's prominence in drug discovery.^54^ Synthesis typically involves oxidation, lacking documented general methods for convergent synthesis. Because there were few recognized methods and quinazoline-1-oxides have potential applications, creating new synthetic routes for them is an interesting task.^55–59^ Using 1 equiv. of nitrobenzene 1c and 1.1 equiv. of benzo[*d*]isoxazole (1d) in a suitable solvent at a specific temperature in the presence of 5 mol% of catalyst, Pawan S. Dhote *et al.*, conducted the reactions (Scheme 2). Using benzo[*d*]isoxazole, α-oxo gold carbene is trapped, sequential N–O bond cleavage is orchestrated, and C–O and C–N bonds are concurrently formed. There was competition between the addition of heteroatoms within and between molecules to alkynes, as well as between the production of α-imino and α-oxo gold carbenes.^60^

### 2.3. Synthesis of polycyclic dihydroquinazolinones (1h)

**Scheme 3:**
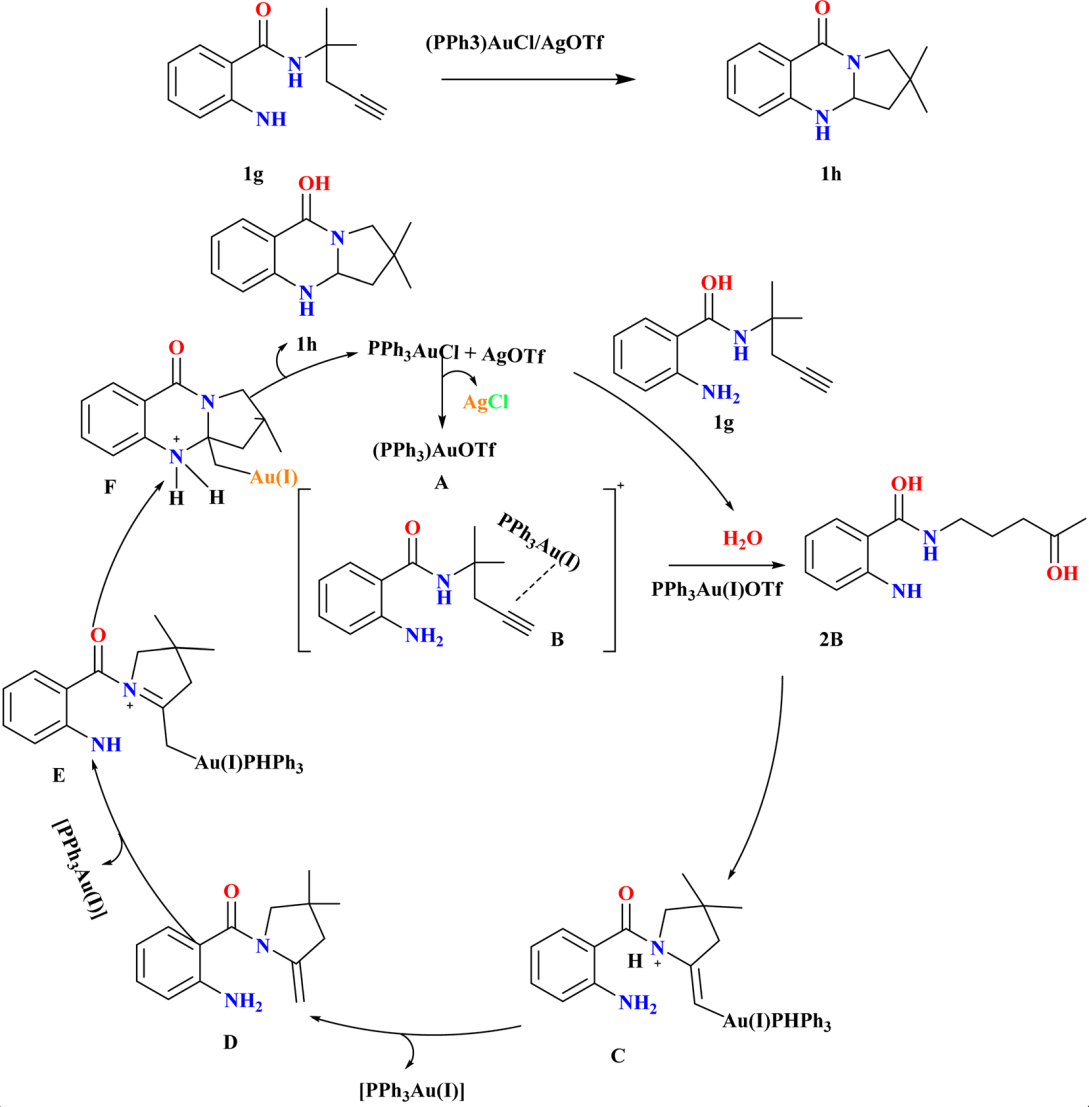
Synthesis and proposed reaction mechanism for the synthesis of 1h at optimized conditions.

Synthesizing polycyclic dihydroquinazolinones from readily available simple substrates under mild conditions remains a challenging yet highly valuable pursuit in organic chemistry.^61–63^ Jingyang Sun *et al.* were synthesized for 1h, in an inert atmosphere, compound 1g and 4 Å molecular sieves (MS) were combined in a flask, followed by the addition of (PPh_3_)AuCl (10 mol%) and AgOTf. After stirring in anhydrous DCE at room temperature for 1.5 hours, the reaction mixture was filtered, and the resulting residue was purified to obtain 1h (85%) as a white solid (Scheme 3). Using (PPh_3_)AuCl/AgOTf at room temperature, the double cascade cyclization of alkyne-tethered anthranilamides exhibited broad substrate scope and functional group compatibility, yielding dihydroquinazolinones in high yields. Both terminal and internal alkynes smoothly underwent cyclization, with even substrates containing fused-aromatic substituents providing excellent yields. Additionally, while phenyl-substituted internal alkynes required higher temperatures for cyclization, they still produced the desired products, mainly favoring 5-*exo*-dig cyclization over 6-*endo*-dig cyclization. Based on literature^64^ according to the suggested process, the active gold-catalyst A was produced by scavenging chloride ions during the condensation of the gold–chloride complex precursor with AgOTf. When A coordinated with the alkyne moiety of the substrate, gold π-alkyne complex B was formed. This complex can then be hydrated to create ketone 2B or cyclized to form gold-alkyl complex C. Active catalyst A was renewed by the protodeauration of C, which released enamine intermediate D. The synthesis of double-cyclized product 1h and regeneration of the cationic gold catalyst A was facilitated by the re-coordination of A to enamine intermediate D, which in turn helped the second intramolecular cyclization.^65^

### 2.4. Diverse synthesis of quinazoline analogues (1j–l)

**Scheme 4:**
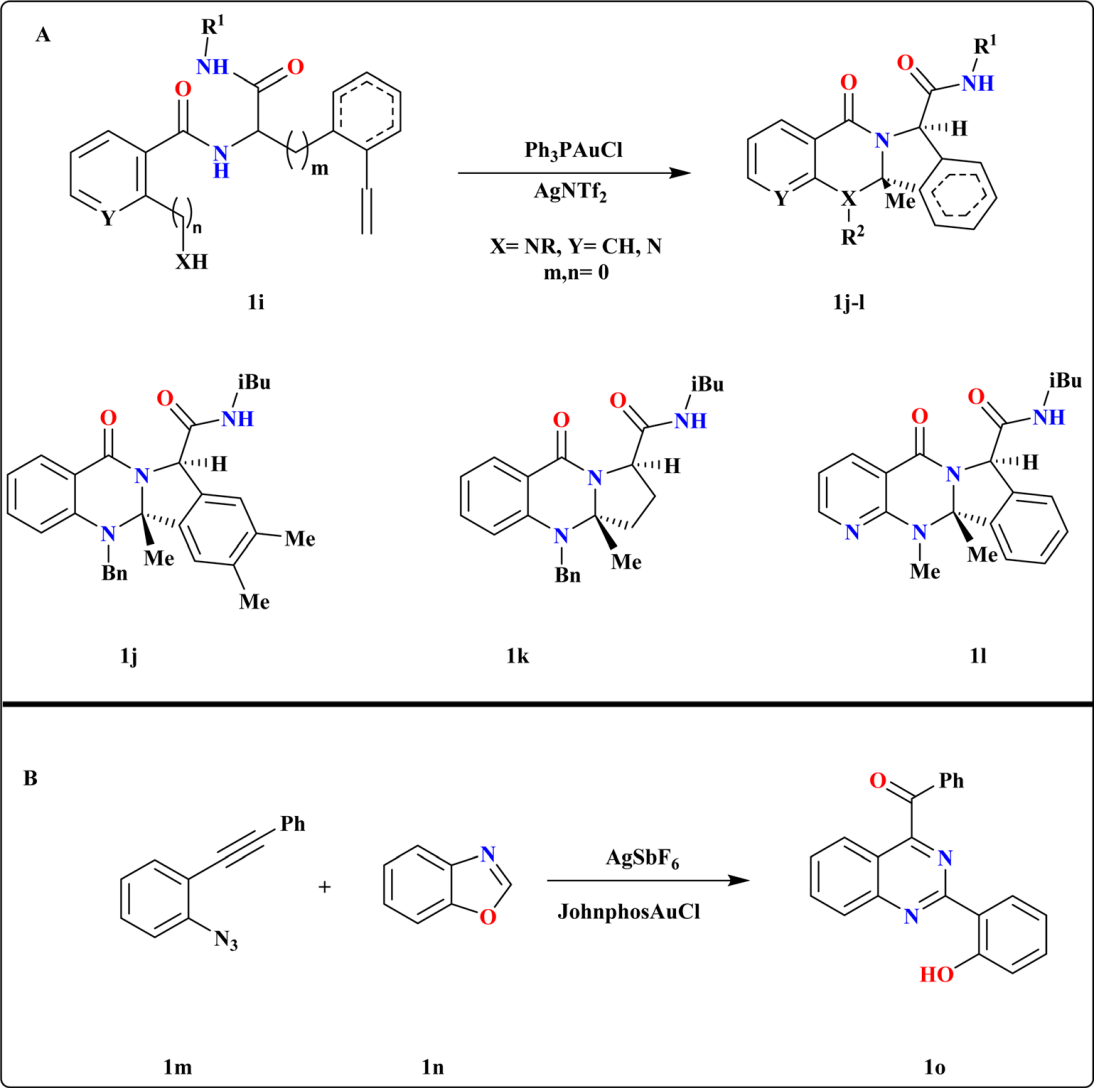
(A) Divergent synthesis of quinazolines analogues (1j–1l) under optimized reaction conditions. (B) Synthesis of 1o, optimized reaction conditions.

Chao Liu *et al.*, initiated their investigation by utilizing 1i as the model substrate, synthesized *via* Ugi-4CR^66^ of 2-ethynylbenzaldehyde, ammonia, salicylic acid, and *tert*-butyl isocyanide. They conducted screening of various Au catalysts, determining that *in situ* generated Ph_3_PAuNTf_2_ yielded the best results (Scheme 4A). Subsequent experimentation revealed that employing Ph_3_PAuCl with chloride scavengers like AgOTf and AgBF_4_ led to reduced yields, with AgNTf_2_ identified as the most efficient catalyst. Substrates derived from 2-ethynyl benzaldehyde, which include an electron-donating dimethyl group, as well as those derived from pent-4-ynal successfully produced quinazolinone analogues 1j–k with impressive yields ranging from 97% to 99%. Additionally, substrates originating from 2-(methylamino)nicotinic acid were also effective in this reaction, yielding quinazolinones 1l at 99%.^67^

### 2.5. Synthesis of (2-(2-hydroxyphenyl)quinazoline-4-yl)(phenyl)methanone (1o)

The investigators were focused on exploring the reaction between 1-azido-2-(phenylethynyl)benzene (1m) and 1,2-benzisoxazole (1n) and screened several phosphine ligands/NHC Au carbene complexes, along with various silver additive combinations (Scheme 4B). Encouragingly, we observed a significant improvement in the yield of 1o to 78% when employing the JohnphosAuCl and AgSbF_6_ combination. Additionally, their investigation into solvent selection revealed that neither nonpolar nor highly polar solvents yielded satisfactory results.^68^

## 3. Gold catalyzed quinoline reactions

### 3.1. Synthesis of tetrahydroquinolines (2c)

**Scheme 5:**
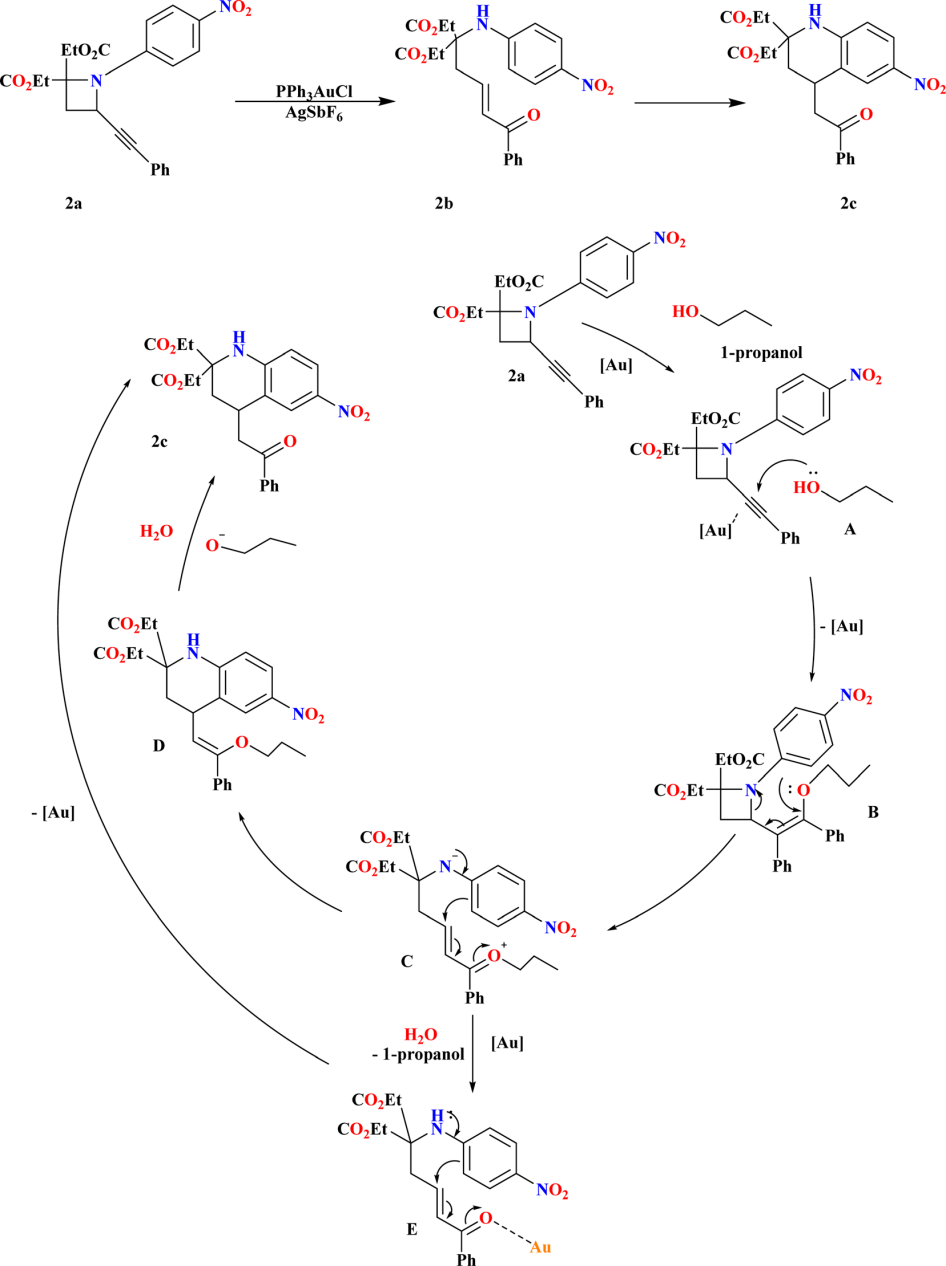
Experiments for mechanistic consideration and proposed reaction mechanism for 2c.

A ring-opening reaction with alcohols facilitated by Au has been discovered as a result of recent attention being paid to the reactivity of 2-alkynylazetidines. This reaction produces δ-amino-substituted α,β-unsaturated ketones.^69–71^ When *N*-4-nitrophenyl-substituted 2-alkynyl azetidines (2a) were heated, according to Touya Kariya *et al.*, an unanticipated cascade reaction occurred, creating 2c by intramolecular Friedel–Crafts-type hydroarylation and Au-promoted ring-opening of the azetidine ring^72,73^ in a single step (Scheme 5). The first coordination of a gold complex to the alkynyl moiety resulted in the formation of the gold–alkyne complex A, which causes the cascade reaction of 2a to 2c. With the help of this complex, alcohol may be added nucleophilically to generate enol ether B. Next, the azetidine ring can be opened, allowing for the intramolecular Friedel–Crafts type conjugate addition to form enol ether D. D hydrolyzes to produce 2c. As an alternative, δ-amino-α,β-unsaturated ketone E was produced by hydrolyzing intermediate C with water in the reaction system. This ketone then passes through Au-promoted intra-molecular hydroarylation to form product 2c.^70^

### 3.2. Synthesis of C2-amidated quinolines (2f)

**Scheme 6:**
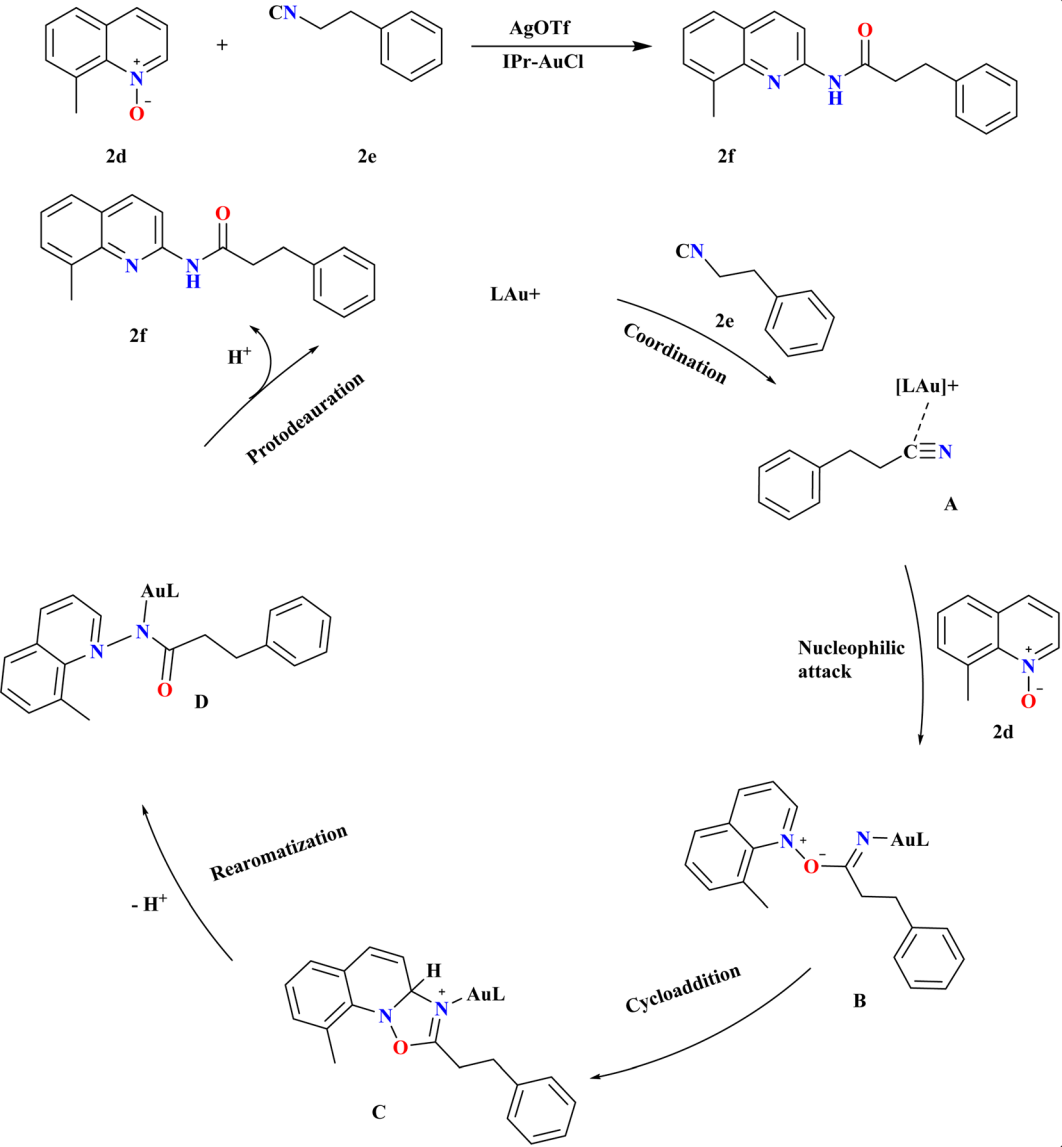
Plausible reaction pathway and optimized reaction conditions for the synthesis of 2f.

The efficient synthesis of *N*-acylated 2-aminoquinolines addresses the demand for diverse functionalized compounds in pharmaceuticals and materials chemistry.^1,74–81^ Recent methods utilizing ionic liquids or ester-radical methyl carbamate offer improved substrate scope and milder conditions compared to traditional approaches.^82–87^ These advancements facilitate the construction of highly functionalized (quinolinyl)amides from quinoline *N*-oxides and nitriles.^88^ Wu, Jiawen *et al.*, studied a gold-catalyzed redox-neutral reaction between 8-methyl quinoline *N*-oxide (2d) and 3-phenyl propane nitrile (2e) yielded *N*-acylated 2-aminoquinoline (2f) in high yields (Scheme 6). IPr ligand gold catalyst and AgOTf co-catalyst in THF at 120 °C for 18 h, providing an almost stoichiometric yield. MeDalphos-AuCl and IPr-AuCl catalysts also proved effective, producing 2f in 70% and 90% yield, respectively. The Au-catalyzed redox-neutral reaction begins with σ-coordination of the Au cation catalyst to the N atom of nitrile 2e, forming intermediate A. Nucleophilic attack by 2dA leads to B, which undergoes intermolecular cycloaddition to form oxazolidine C. Ring-opening and aromatization yield amidated intermediate D, culminating in the desired C2-amidated quinoline 2f after protodeauration, a core structure in various bioactive molecules, with good functional group tolerance and simple steps.^89^

### 3.3. Synthesis of 3-alkoxy-4-quinoline (2h)

**Scheme 7:**
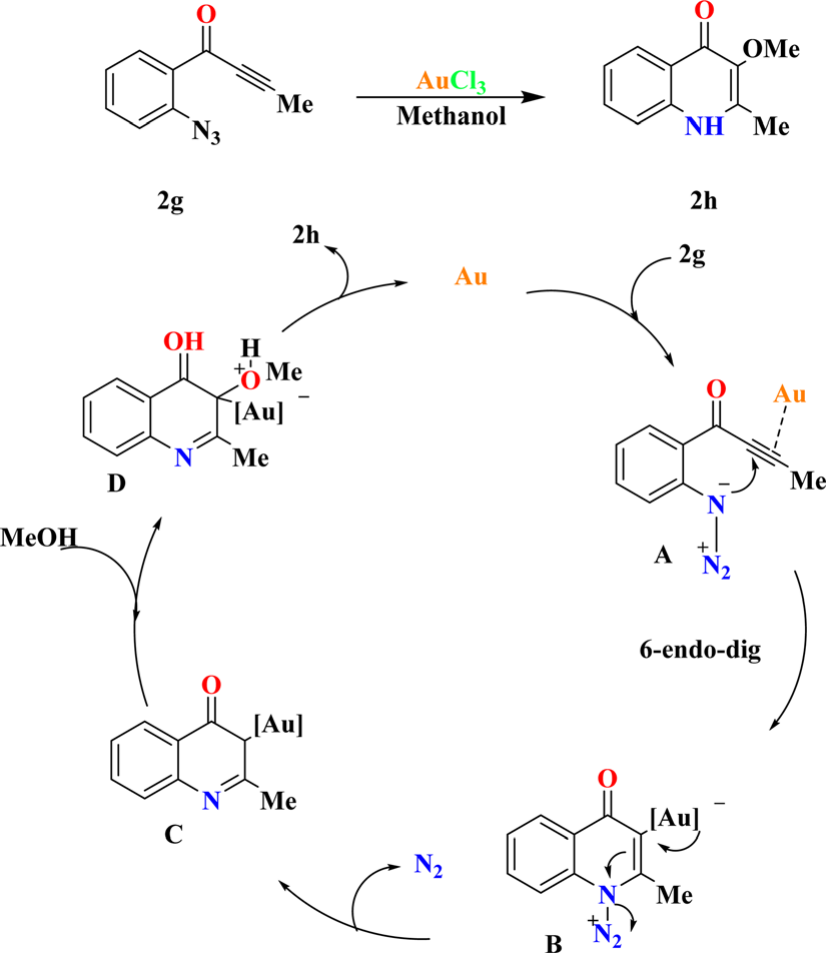
At optimized conditions proposed reaction mechanism for synthesis of 2h.

The fact finders synthesized 2h by the reaction of ynones (2g) (0.2 mmol) and AuCl_3_ (5.0 mol%) in methanol (1.0 mL), achieving a 95% yield of 2h. XPhosAu(CH_3_CN)NTf_2_ exhibited lower reactivity at room temperature (50% conversion), requiring 80 °C to achieve comparable reactivity (58% yield of 2h) with byproduct contamination. Drawing from prior studies and relevant literature sources,^90–92^ a plausible mechanism is proposed in Scheme 7. Initially, 6-*endo*-dig cyclization of the gold-activated alkyne group with the tethered azide moiety produces the adduct A, which then yields the crucial α-imino gold carbene species B upon N_2_ extrusion. Subsequent trapping of the gold carbene intermediate by external alcohol yields the O–H insertion products 2h, regenerating the catalyst.^93^

### 3.4. Synthesis of pyrrolo[1,2-*b*]isoquinolines (2j)

**Scheme 8:**
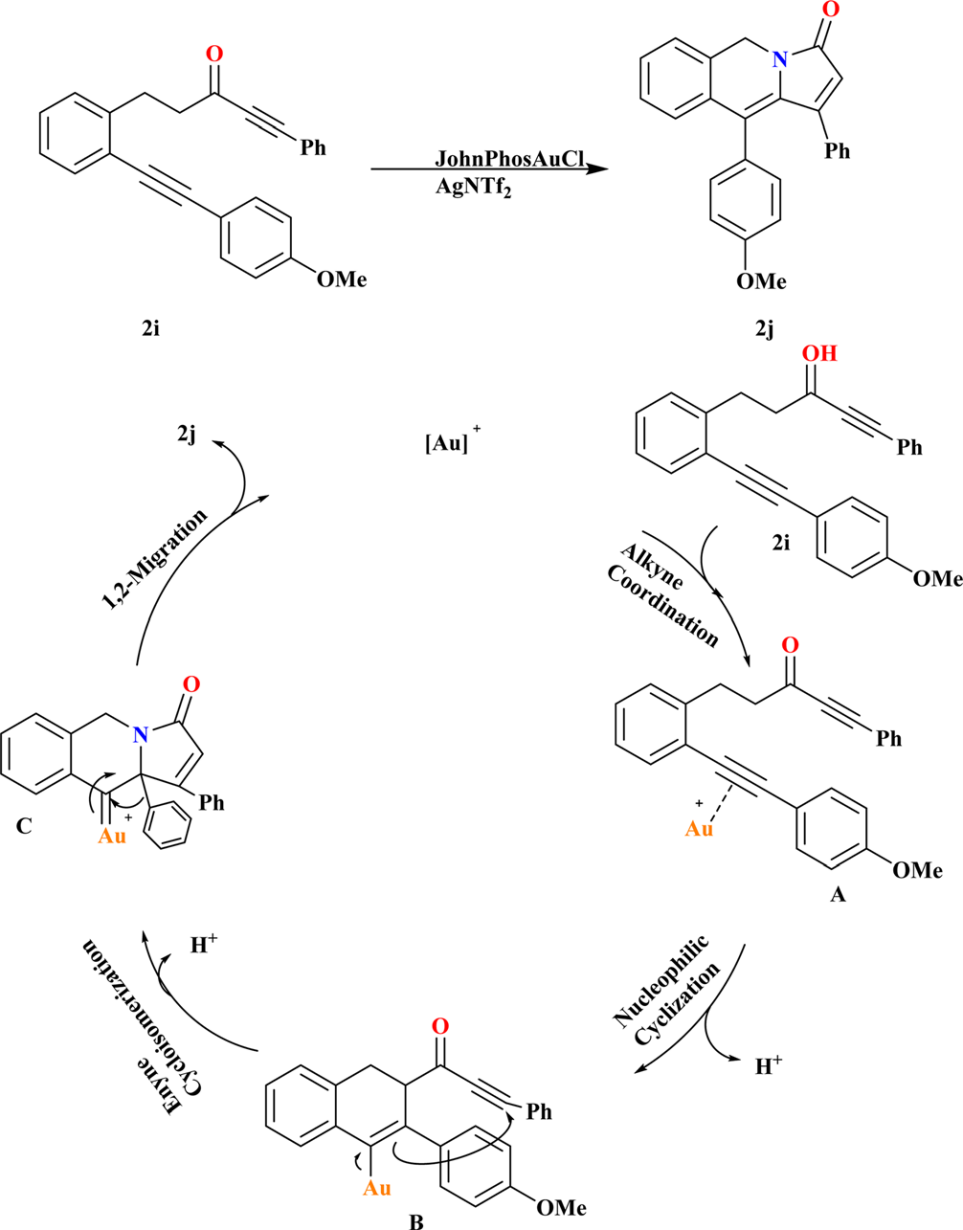
Synthesis and possible reaction mechanism of 2j at optimized conditions.

In the results of gold-catalyzed 1,2-aryl migration of 2i, it was found that *in situ* generated JohnPhosAuOTf is the most effective catalyst investigated by Liangliang and his coworkers. Using JohnPhosAuCl with different chloride scavengers, including AgNTf_2_, AgSbF_6_, AgBF_4_, or Ag_2_CO_3_, demonstrated that AgNTf_2_ is the optimal choice. Gratifyingly, the reaction proceeded smoothly at room temperature, affording product 2j an 89% yield (Scheme 8). Based on literature,^94–98^ a plausible mechanism for the gold-catalyzed transformation of 2i is proposed. Initially, substrate 2i undergoes alkyne coordination followed by nucleophilic cyclization, forming intermediate B*via* a six-*endo*-dig pathway. Cyclo-isomerization of B leads to the formation of gold carbenoid intermediate C. Finally, migration of the alkyl or aryl group results in the generation of product 2j, liberating the gold-catalyst for subsequent cycles.^99^

### 3.5. Synthesis of tetrahydrobenzo[*g*]quinolines (2l)

**Scheme 9:**
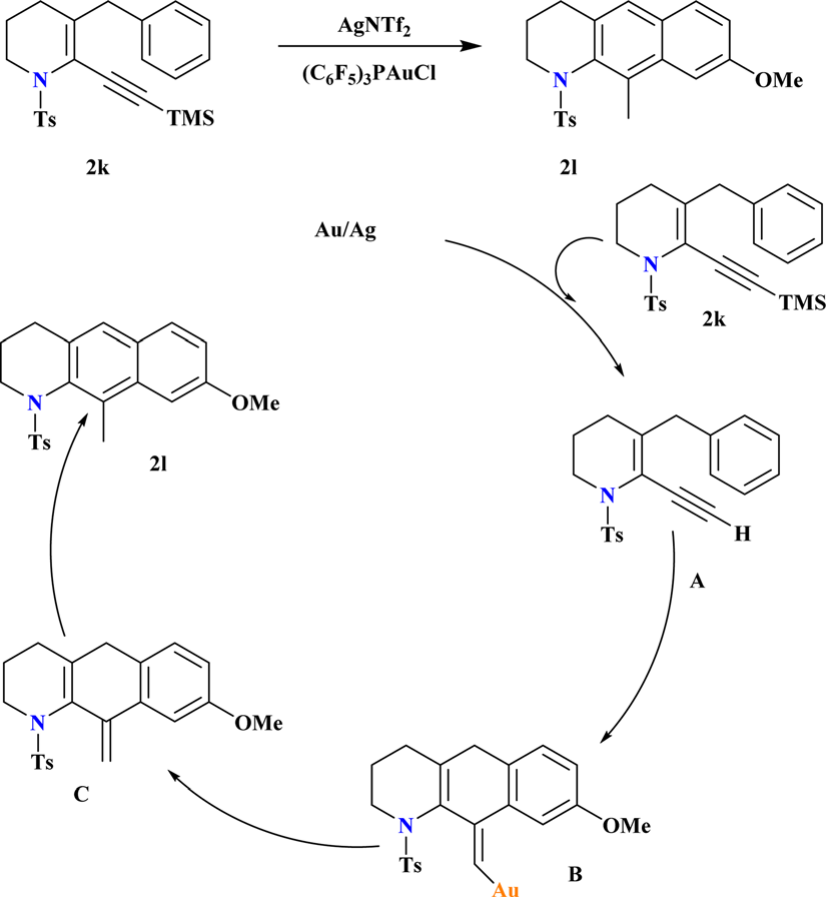
Synthesis and possible reaction pathways for production of 2i.

A novel catalyst (C_6_F_5_)_3_PAuCl, was synthesized and known for its efficacy in the hydroarylation of *o*-propargyl biaryls (2k). Combining this electron-poor ligand with AgNTf_2_ significantly enhanced the yield of 2l, while AgOTf was less effective (Scheme 9). In another study,^100^ Ye *et al.*, demonstrated that incorporating the strong Brønsted acid HNTf_2_ into the reaction medium improved the final yield of gold-catalyzed synthesis of anthracenes. Reaction conducted without air, moisture exclusion, and chemo selective over triple bond hydration in the non-anhydrous solvent. The proposed hydroarylation mechanism involves initial desilylation of the alkyne, observed through conversion of 2k to A. Subsequent reaction of A under Au-catalyzed conditions yields comparable yields of the hydroarylation product. The vinyl gold intermediate B undergoes protodeauration to form *exo* intermediate C, which aromatizes rapidly to yield the final product 2l.^101^

### 3.6. Synthesis of C3-indolyl quinoline (2o)

**Scheme 10:**
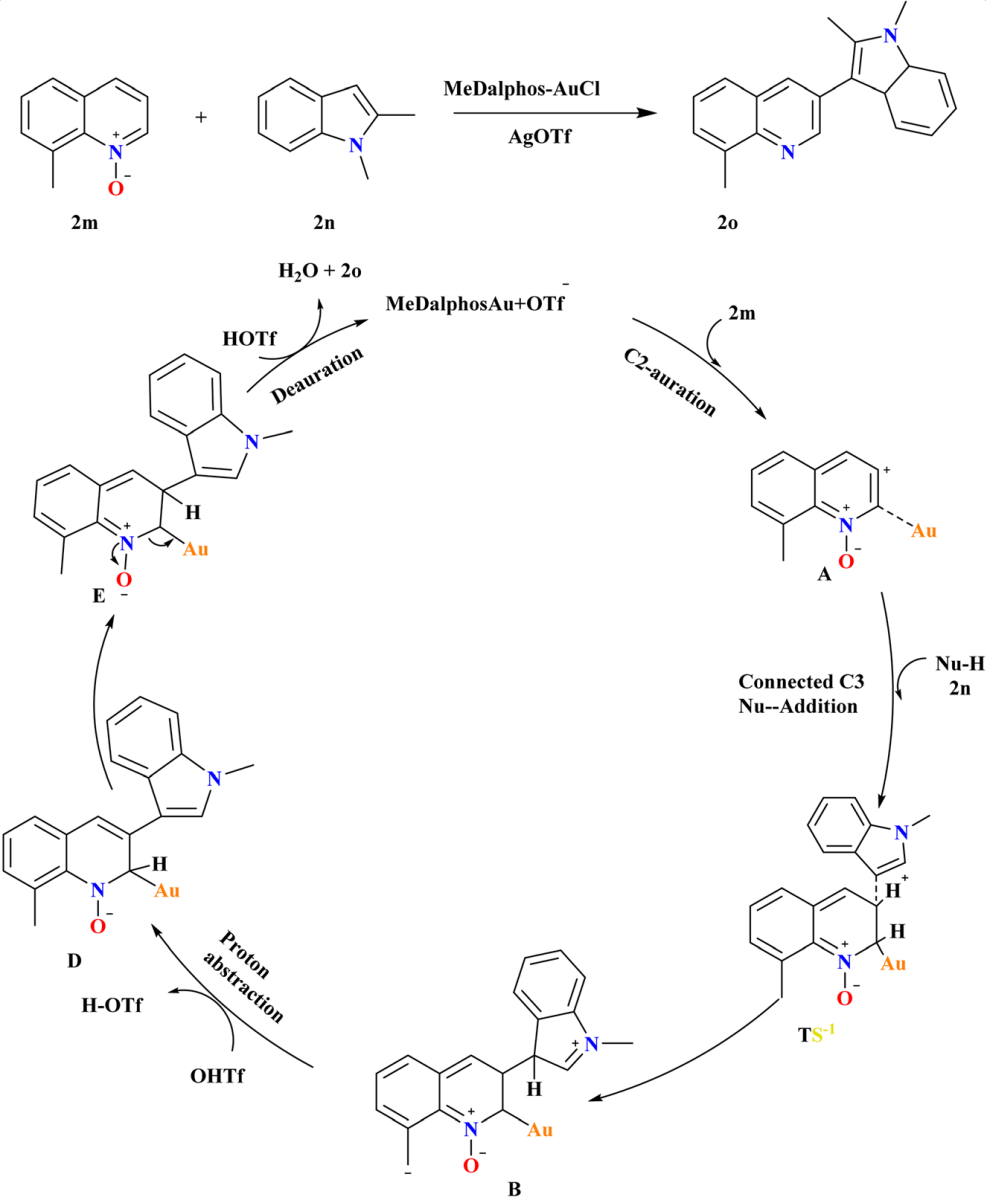
Synthesis and possible reaction pathways for production of 2o.

8-Methyl quinoline *N*-oxide (2m) and 1,2-dimethyl-1*H*-indole (2n), were employed at various reaction conditions and the desired product 2o was achieved with a 99% yield using MeDalphosAuCl catalyst (5 mol%) combined with AgOTf co-catalyst (10 mol%) in MeCN at 120 °C for 18 h (Scheme 10). Ph_3_PAuCl and SIPrAuCl catalysts were also effective but less so, yielding 2o at 58% and 56%, respectively but for the synthesis of indole derivatives it was more effective and showed excellent yield.^102^ A proposed mechanism for Au-catalyzed selective C3–H functionalization of quinoline *N*-oxides involves C2-auration forming *ortho*-Au(i)-activated intermediate A, facilitating nucleophilic C3 attack. This leads to TS-1, promoting C–C coupling to form B. AgOTf counter anion assists in proton abstraction from indole 2n and 2m, yielding C and D, respectively. Subsequent deauration generates the desired C3-substituted quinoline product 2o and H_2_O, closing the catalytic cycle.^88^

### 3.7. Synthesis of 3-sulfonyl quinolines (2r)

**Scheme 11:**
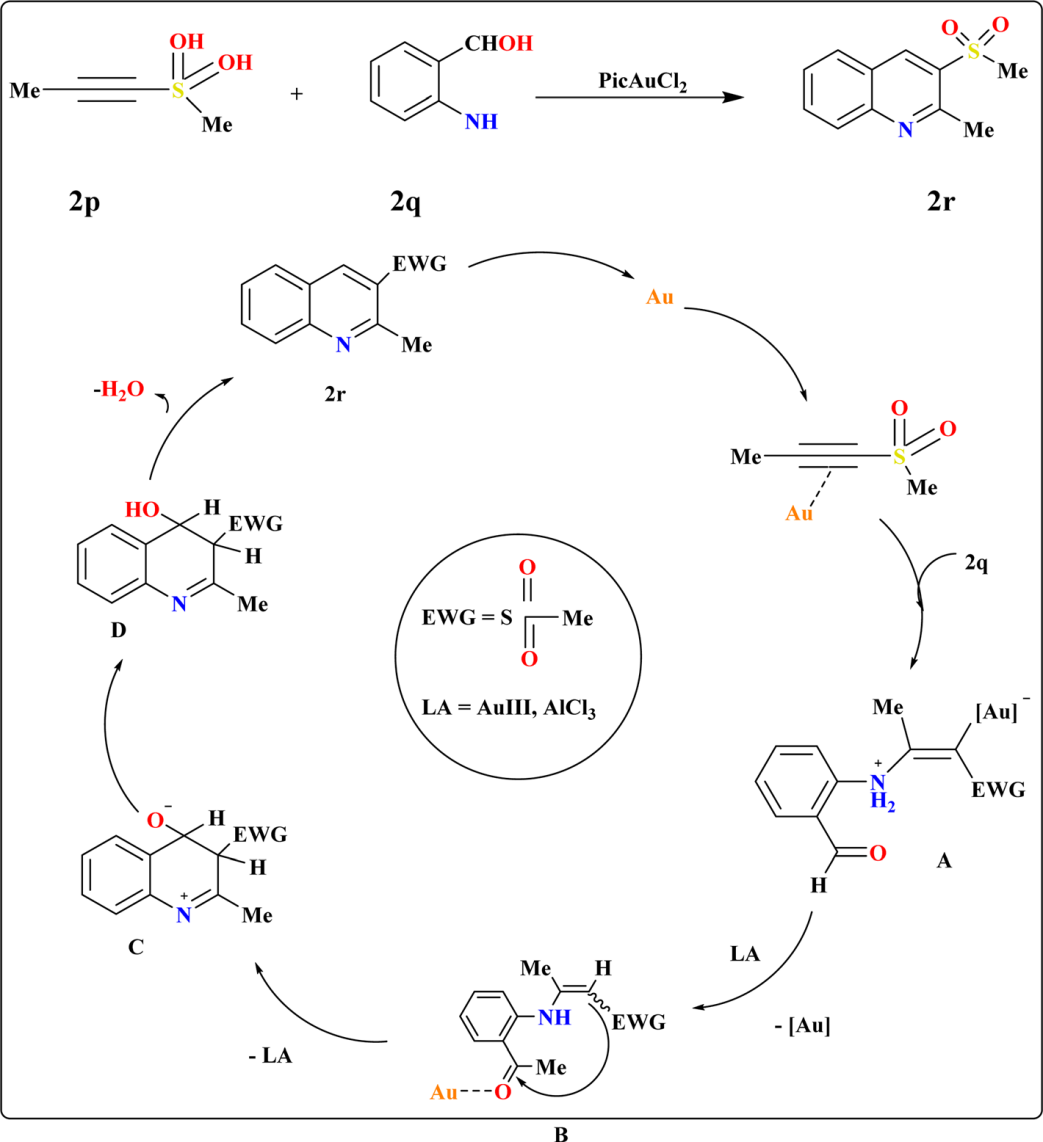
Synthesis and possible reaction route for production of 2r.

The Friedlander synthesis (FS) enabled the one-step preparation of 3-substituted quinolines from diverse starting materials.^103^ Wang *et al.*, proposed an improved method for synthesizing 3-sulfo-quinolines, addressing previous challenges with no selectivity and low yields.^104,105^ Recent findings demonstrate alkynyl sulfones as β-keto sulfone substitutes due to regioselectivity in reactions, particularly under mild conditions facilitated by gold complexes.^23,106–109^ Elena I. *et al.*, suggested a plausible alternative to FS for modular one-step synthesis of 3-sulfonylquinolines (2r) using alkynylsulfones (2p) and 2-aminobenzaldehyde (2q) (Scheme 11). In testing their hypothesis, they examined the reaction between 2p and 2q to produce 2r under varied conditions. Au(iii) complexes emerged as the most efficient catalysts,^110^ with the highest yield of 2r achieved using 5 mol% PicAuCl_2_ in DCE at 60 °C for 3 h, supplemented with 4 Å molecular sieves to capture released water. Gold-catalyzed conditions were successful in annulating various electron-deficient alkynes, yielding diversely substituted quinolines at position 3. The proposed mechanism suggests a dual role for the gold-catalyzed-based catalyst, activating both C–C bonds and carbonyl groups to facilitate hydroamination and subsequent cyclization.^111^

### 3.8. Synthesis of quinoline-3-ylphosphonate (2u)

**Scheme 12:**
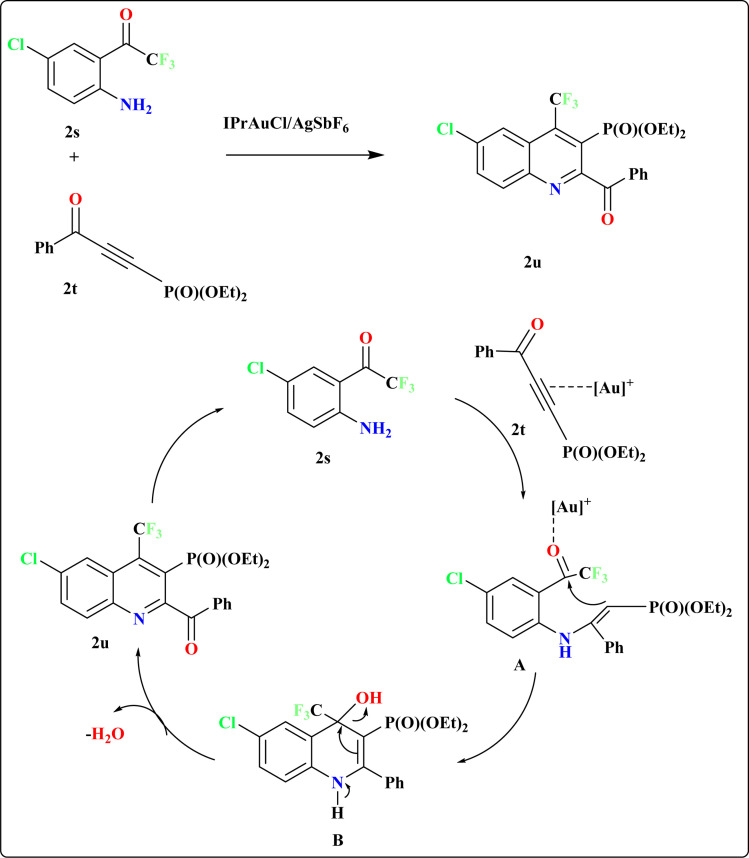
Synthesis and proposed reaction mechanism for 2u at optimized conditions.

Recently, researchers have introduced a regio-divergent approach for synthesizing quinolines^112^ with CF_3_ and P(O)(OEt)_2_ groups, achieved through the reaction between 2′-amino-2,2,2-trifluoroacetophenones (2s) and (3-oxoprop-1-yn-1-yl)-phosphonates (2t) catalyzed by and IPrAuCl/AgSbF_6_ catalyst (Scheme 12). The mechanism underlying this regio-divergent process remains unknown. Shifts in electron density on the alkyne likely facilitate selective attack by the amino group of compound 2s. Subsequent cyclization of the intermediate hydroamination product, followed by elimination of water, results in the formation of quinoline 2u.^113,114^

### 3.9. Synthesis of 4-(1*H*-pyrrol-2-yl)quinoline (2x)

**Scheme 13:**
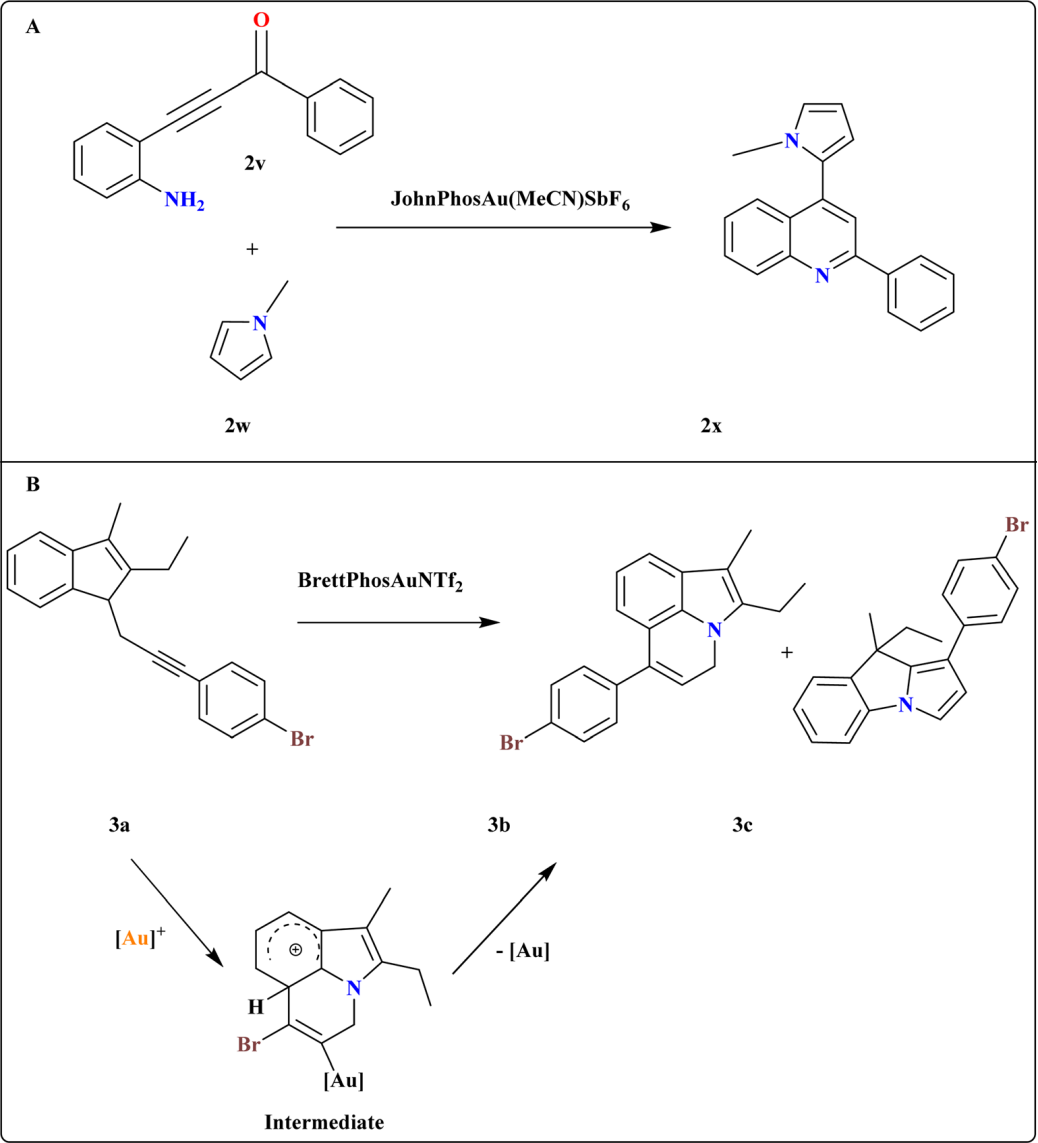
(A) Synthesis of 2x at optimized reaction conditions. (B) Synthesis and catalyst-controlled divergent cycloisomerization at optimized conditions.

A novel method developed for synthesizing 2x*via* sequential regioselective direct heteroarylation/cyclocondensation reactions of β-(2-aminophenyl)-α,β-ynones (2v) with pyrrole derivatives (Scheme 13A). The reaction of 2v with 1-methyl-1*H*-pyrrole (2w) in DCE at 60 °C with 1 equiv. of TfOH and an excess of 3 equiv. of 2w, together with 5 mol% of the JohnPhosAu(MeCN)SbF_6_ catalyst, was carried out under ideal circumstances. In 2w, the most important factors influencing the selectivity of the C–H functionalization site were temperature, reaction medium, and catalyst characteristics.^115^

### 3.10. Synthesis of 4*H*-pyrrolo[3,2,1-*ij*]quinoline (3b)

A catalyst-controlled divergent cycloisomerization of indolyl-ynes, yielding complex 9*H*-pyrrolo[1,2-*a*]indoles (3c) and 3b from *N*-propargyl indole substrates (3a) as shown in Scheme 13B. Initial screening using various Au catalysts showed Ph_3_PAuNTf_2_ (ref. 116) as effective, yielding products 3b and 3c in 65.5% total yield. While the steric bulky and electron-rich Buchwald-type ligand^117^ BrettPhos increased both total yield (71%) and selectivity, the N-heterocyclic carbene ligand IPr^118^ produced a comparable total yield with somewhat better selectivity. To explain the chemo-vergence in the cycloisomerizations of 3a that are catalyzed by platinum and gold, a reasonable mechanism was put forward. The cationic [BrettPhosAu]^+^ activated acetylenic link in substrates with 2,3-substitution and 7-unsubstitution favors the sterically less hindered 7-position for initial addition, minimizing steric repulsion with bulky ligands.^119^

### 3.11. Synthesis of indolo[1,2-*a*]quinolin-5(6*H*)-ones (3g)

**Scheme 14:**
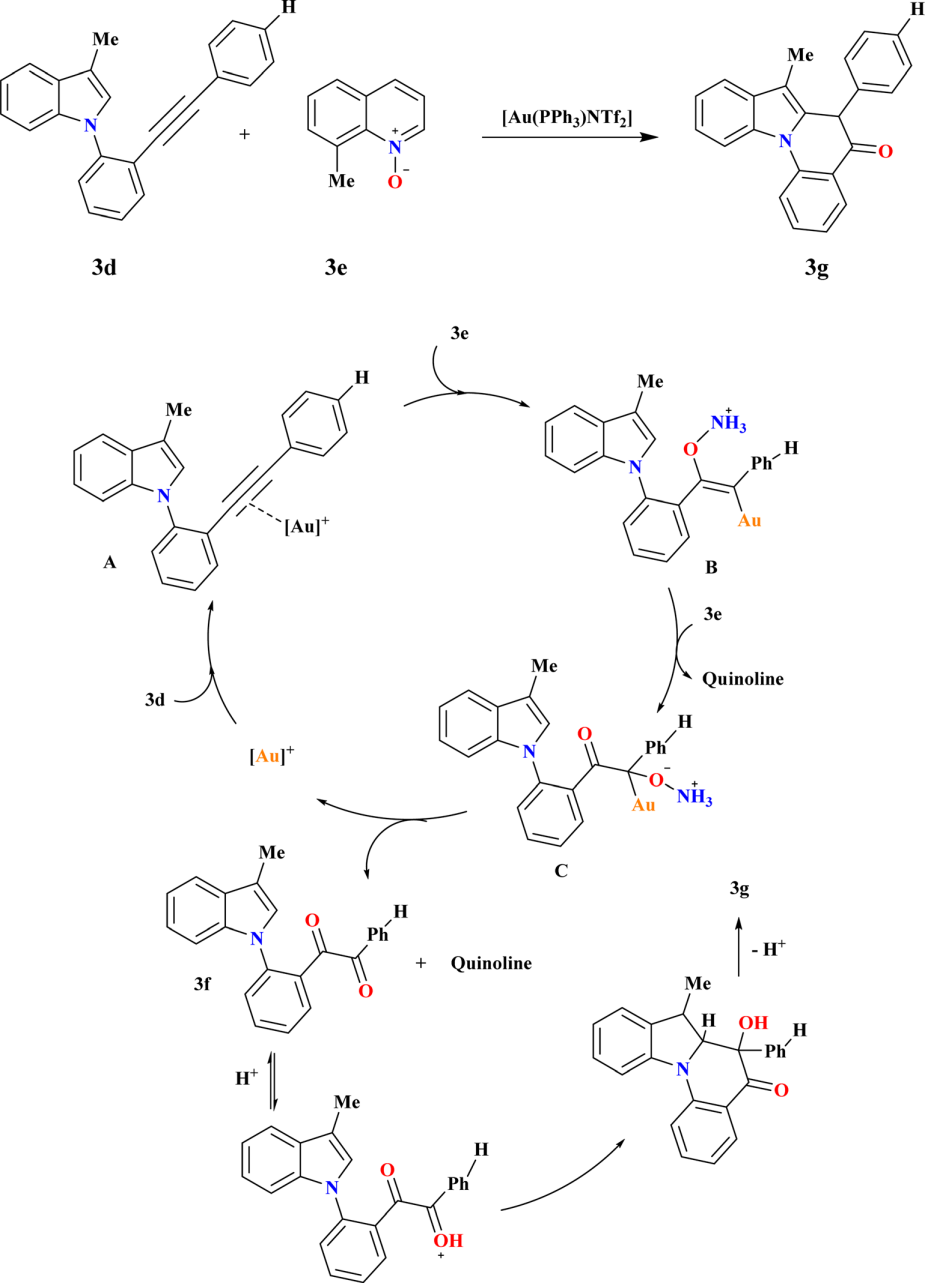
Synthesis and proposed reaction pathways for 3g at optimized reaction conditions.

The compound 3f was synthesized in excellent yields by utilizing the amount of 3e to 2.5 equiv. at a reaction temperature of 65 °C, 3g was obtained with an overall yield of 87% (Scheme 14). To further enhance the yield of 3g, after complete conversion of 3d (confirmed by TLC analysis after 3 hours at 65 °C), a solution of HCl in cyclopentyl methyl ether (CPME) was introduced to the reaction mixture to promote the cyclization step. Following an additional 0.5 hours at room temperature, 3g was isolated in 87% yield. Based on observed reactivity and prior literature,^120–124^ initially, coordination of the gold-catalyst to the triple bond of indole derivative 3d forms intermediate A, which undergoes nucleophilic addition of 3e to generate vinyl gold intermediate B. Subsequent attack by another molecule of 3e yields intermediate C and quinoline. Elimination of a second quinoline molecule and deauration result in the formation of 1,2-dicarbonyl derivative 3f. Cyclization product 3g is obtained *via* acid-promoted nucleophilic addition of indole C2 carbon to the distal carbonyl of indole-1,2-dione 3f, followed by re-aromatization.^125^

### 3.12. 1-Benzyl-1,2,3,4-tetrahydroquinoline (3i)

**Scheme 15:**
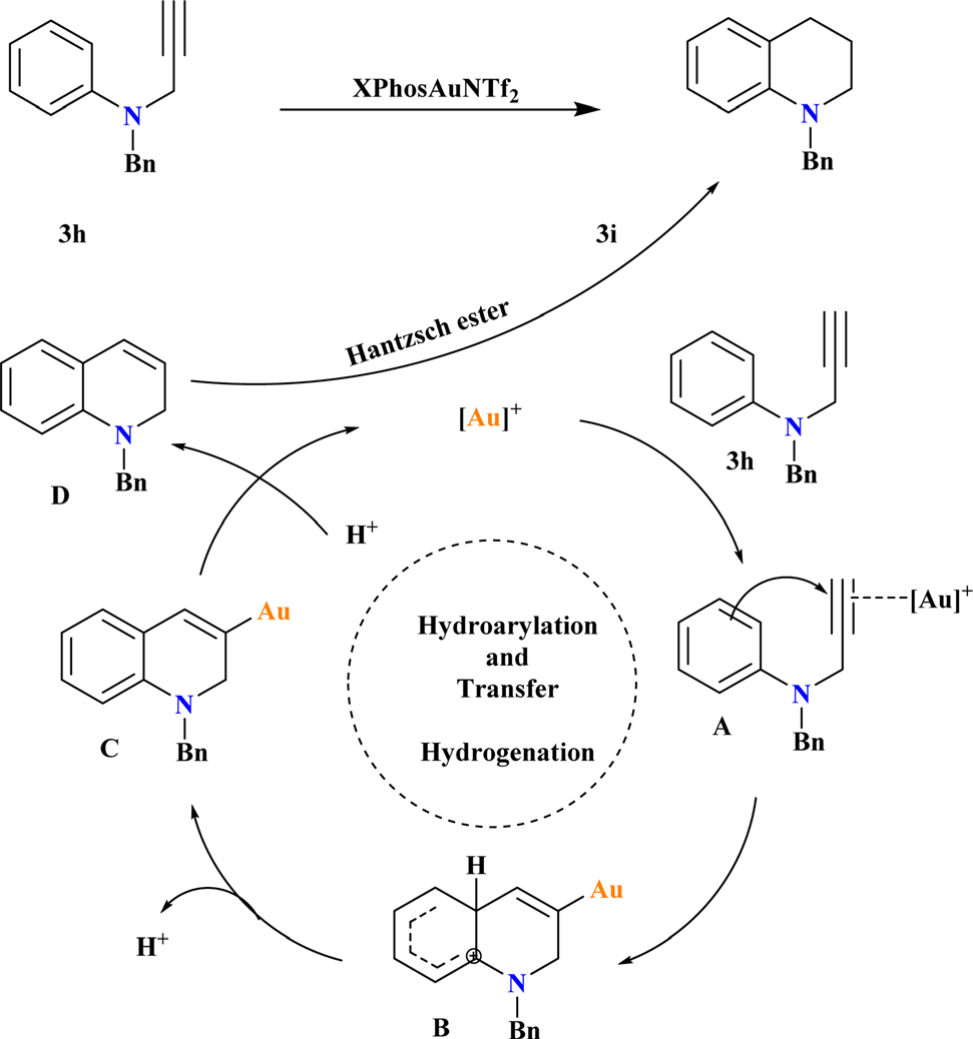
Synthesis and possible reaction mechanism for 3i at optimized conditions.

A new Au-catalyzed protocol was developed for synthesizing tetrahydroquinolines from *N*-aryl propargyl amines using tandem intramolecular hydroarylation^72^ and transfer hydrogenation reactions. After testing various conditions, the researcher optimized the reaction to achieve the highest yield by conducting it in a sealed tube at 65 °C for 24 h under a nitrogen atmosphere, using 1 equivalent of 1a, 1.5 equivalents of HEH, and 5 mol% of XPhosAuNTf_2_ in HFIP (Scheme 15). They found that lowering the reaction temperature below 65 °C led to a decrease in yield, which stabilized at this temperature. Based on previous studies^126–128^ the proposed mechanism involves the formation of complex A through the η^2^-coordination of the alkyne moiety with the Au^+^ catalyst. Electrophilic aromatic substitution yields intermediate B, which undergoes deprotonation to form complex C. Protonation of complex C leads to intermediate D, which is reduced by Hantzsch ester to yield the target product 3i.^129^

### 3.13. Gold-catalyzed hydrogenation of quinolines

**Scheme 16:**
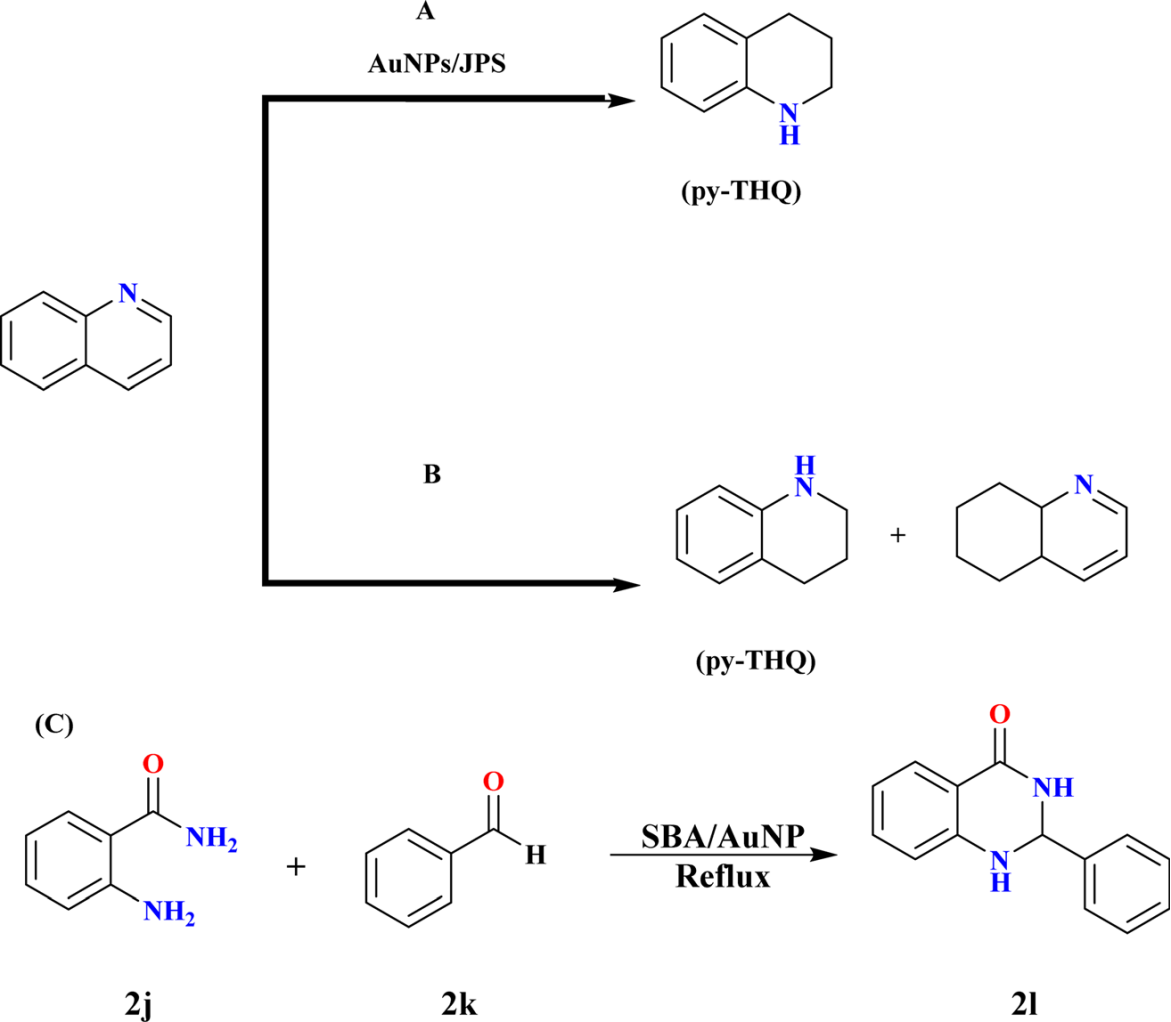
(A) The hydrogenation of quinoline using AuNPs/JPS. (B) Hydrogenation of quinoline into py-THQ at optimized reaction conditions. (C) Synthesis of 2l at optimized conditions.

Jute plant stems (JPS) can be used as a support for Au nanoparticle (AuNP) synthesis without the need for external reducing chemicals, according to a technique presented by Adeyeye Nafiu Sodiq *et al.*; these nanoparticles showed that they were capable of reducing quinolines by catalysis. The hydrogenation of quinoline under molecular hydrogen pressure was used to test the AuNPs/JPS catalyst's broader applicability. As seen in Scheme 16A, the hydrogenation of quinoline to get 1,2,3,4-tetrahydro quinoline has attracted a lot of attention lately because of its applicability in the production of agrochemicals, medications, dyes, and other alkaloids.^130,131^ Ren *et al.*; achieved remarkable results by demonstrating the peculiar chemo-selective hydrogenation of quinoline compounds utilizing a gold-supported catalyst. The model reactant utilized in this work to assess the activity and selectivity of hydrogenation at molecular hydrogen pressure was quinoline.^132^ Dichloromethane (DCM) and NaBH_4_ were two of the solvents used for hydrogenation, as activity and selectivity rely on the solvent. Quinoline was hydrogenated in a Teflon-lined autoclave equipped with mechanical stirring, temperature control, and pressure monitoring. Quinoline, anhydrous DCM, and the AuNPs/JPS catalyst (5 mol%) were supplied to the reactor under frequent hydrogen flushing, and it was subsequently pressurized with 30 bars of H_2_. At 100 °C, the reaction was continuously stirred for 20 hours before being cooled and depressurized.^131,133^

For instance, hydrogenation does not take place even after 20 hours of reaction at 50 °C in DCM at 30 bar hydrogen pressure. However, when the temperature is increased from 70 to 100 °C, the conversion improves significantly, producing >99% of the product with the pyridine ring hydrogenated. Yet, when the reaction duration is shortened from 20 hours to 10 hours, the reaction remains incomplete with only 77% conversion. Hydrogen pressure was also optimized during this study.^134^ Quinoline hydrogenation was carried out by Jianbo Zhao *et al.* at 100 °C using a stainless-steel autoclave filled with 3.0 mL of water, 60 μL of quinoline, and 0.1 g of gold catalysts at an H_2_ pressure of 2.0 MPa (Scheme 16B). Following completion, ethyl acetate was used to extract the reaction mixture three times while it was cooled. Comparing the 1.2% Au@SBA-15-500 catalyst to the 1.3% Au/SiO_2_-500 catalyst, the former showed better activity, selectivity towards py-THQ, and remarkable sintering resistance up to 800 °C. The mesopores and small-sized gold nanoparticles of SBA-15 were responsible for these properties, which allowed for significant quinoline derivative adaptability and recyclability.^135–137^

### 3.14. Synthesis of 2-benzyl-2,3-dihydroquinazolin-4(1*H*) ones (2l)

Cezar A. Didó and his co-workers synthesized 2l by reaction of 2-amino benzamide (2j) and benzaldehyde (2k) with 3 mL ethanol and 30 mg SBA/AuNP catalyst (0.6 mol% gold) under reflux until complete consumption of starting materials (Scheme 16C). Catalyst separation *via* filtration followed by washing with hot ethanol, and product crystallization and drying under reduced pressure were adopted.^138^ Product characterization was conducted through NMR analysis in DMSO-d_6_, small gold nanoparticles anchored to SBA-15 *via* a cationic silsesquioxane coating, allowing high dispersion. The optimal gold amount ensures complete anchoring and conversion into nanoparticles during the reduction process. The SBA/AuNP catalyst significantly enhances the yield of 2l with rapid reagent consumption in just 20 minutes. Decreasing catalyst load prolongs reaction time. SBA-15 catalyst without gold nanoparticles yields only 40% of 1; even after 3 hours. Despite a 10% decrease in yield over 3 consecutive runs, the SBA/AuNP catalyst remains effective with a 70% yield in the 3rd recycling run.^139^

## 4. Summary and outlook

The recent advancements in gold-catalyzed cascade protocols for synthesizing quinoid heterocycles, spanning 2020 to 2024, signify a transformative leap in organic synthesis. Looking forward, further exploration and optimization of these protocols could focus on enhancing reaction scope, selectivity, and sustainability. The use of gold-catalyzed cyclization in synthesizing complex quinoid scaffolds, often difficult to achieve through conventional methods, underscores the effectiveness of gold-mediated processes. The rapid advancement of gold-catalyzed reactions in forming quinoid heterocycles presents an opportunity for developing environmentally friendly processes, leading to the production of valuable fine chemicals, natural products, and pharmaceuticals in a sustainable manner. Integrating computational methods could aid in designing novel catalysts and predicting reaction outcomes. Additionally, expanding mechanistic understanding could guide the development of more efficient and predictable synthetic routes. Collaboration between synthetic chemists, computational chemists, and chemical engineers will be crucial for translating these innovations into practical applications. Furthermore, exploring the biological activities of newly synthesized quinoid compounds could uncover novel therapeutic agents. Overall, with continued research and innovation, these advancements hold immense potential for driving progress in both academic and industrial settings, paving the way for the synthesis of diverse quinoid heterocycles with unprecedented precision and efficiency.

## Data availability

No primary research results, software, or code have been included and no new data were generated or analyzed as part of this review.

## Author contributions

Adnan Majeed: writing – original draft, software. Ayesha Zafar: writing – review & editing. Zanira Mushtaq: data curation, validation. Muhammad Adnan Iqbal: conceptualization, resources, supervision.

## Conflicts of interest

The authors declare no conflict of interest.
